# Supplementary material for: A variational deep-learning approach to modeling memory T cell dynamics
Source: bioRxiv. 2025 Feb 25:2024.07.08.602409. Preprint. [Version 2] doi: 10.1101/2024.07.08.602409 (PMC11888226; doi:10.1101/2024.07.08.602409)
Supplement: Supplement 1 [file NIHPP2024.07.08.602409v2-supplement-1.pdf]

bioRxiv preprint doi: https://doi.org/10.1101/2024.07.08.602409; this version posted February 25, 2025. The copyright holder for this preprint (which was not certified by peer review) is the author/funder, who has granted bioRxiv a license to display the preprint in perpetuity. It is made available under aCC-BY 4.0 International license.

Supporting Information

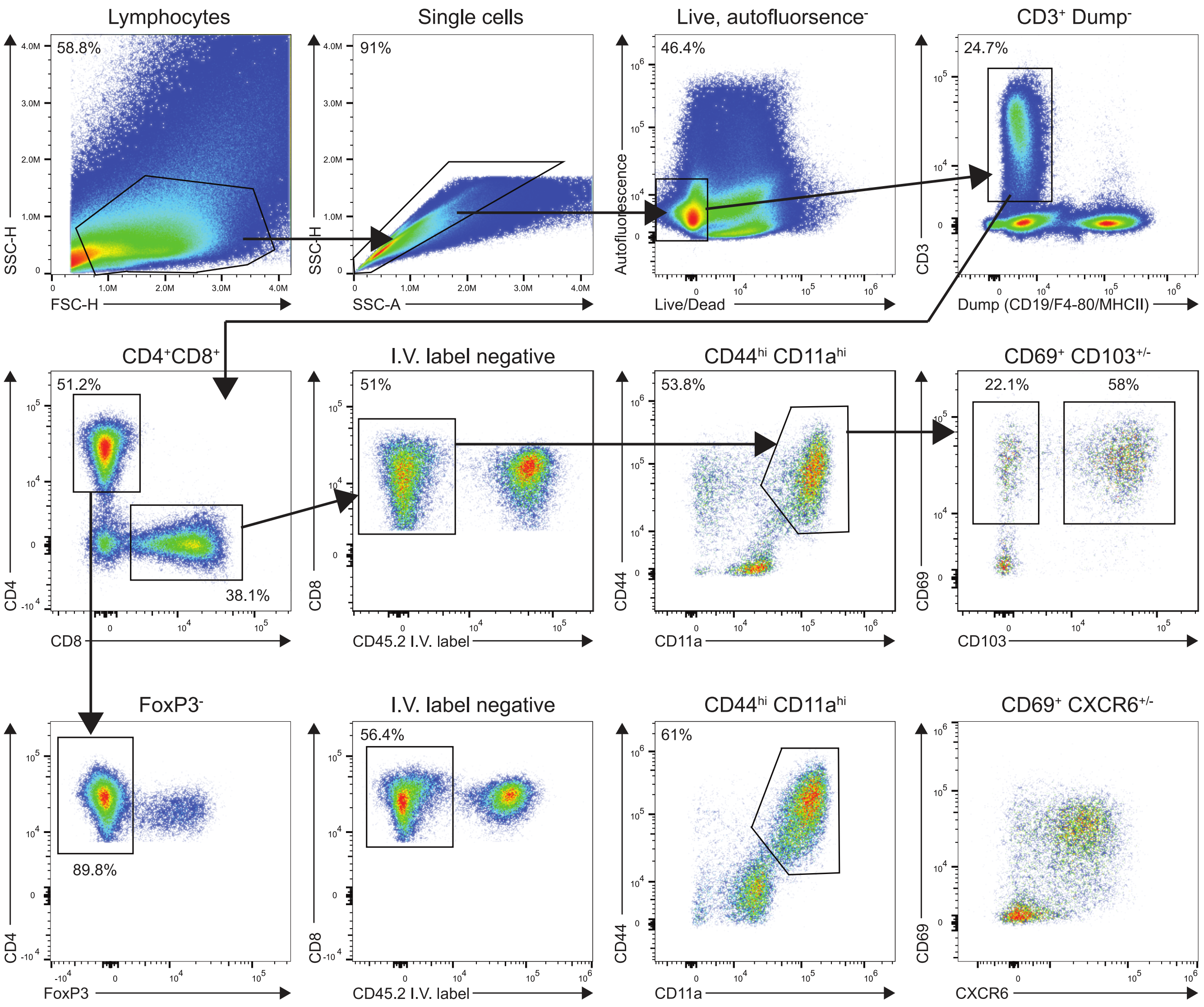

Figure S1: Gating Strategy for CD8$^+$ and conventional CD4$^+$ antigen experienced T cells residing in the lung.

bioRxiv preprint doi: https://doi.org/10.1101/2024.07.08.602409; this version posted February 25, 2025. The copyright holder for this preprint (which was not certified by peer review) is the author/funder, who has granted bioRxiv a license to display the preprint in perpetuity. It is made available under aCC-BY 4.0 International license.

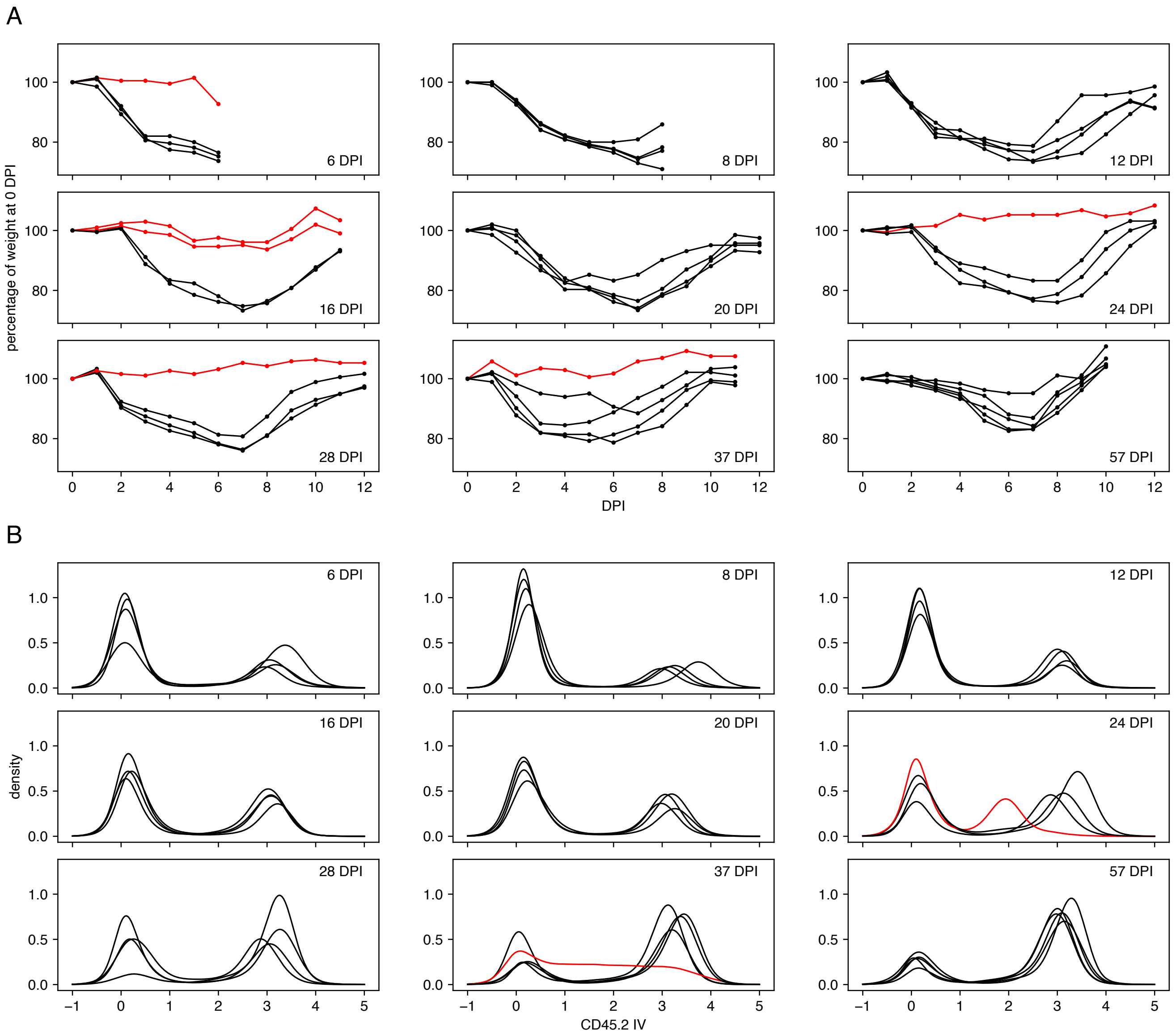

Figure S2: Weight curves of mice after IAV challenge and assessment of I.V. labeling. Each cohort contained 4 or 5 mice. In total data from 38 mice is presented. **A.** The curves indicate the percentage of the weight relative to that on the day of infection. The red curves correspond to mice that were excluded from further analysis due to lack of weight loss. **B.** Distribution of the IV label (CD45.2 IV) for each mouse. The red curves correspond to mice that were excluded from further analysis due to poor IV labeling.

bioRxiv preprint doi: https://doi.org/10.1101/2024.07.08.602409; this version posted February 25, 2025. The copyright holder for this preprint (which was not certified by peer review) is the author/funder, who has granted bioRxiv a license to display the preprint in perpetuity. It is made available under aCC-BY 4.0 International license.

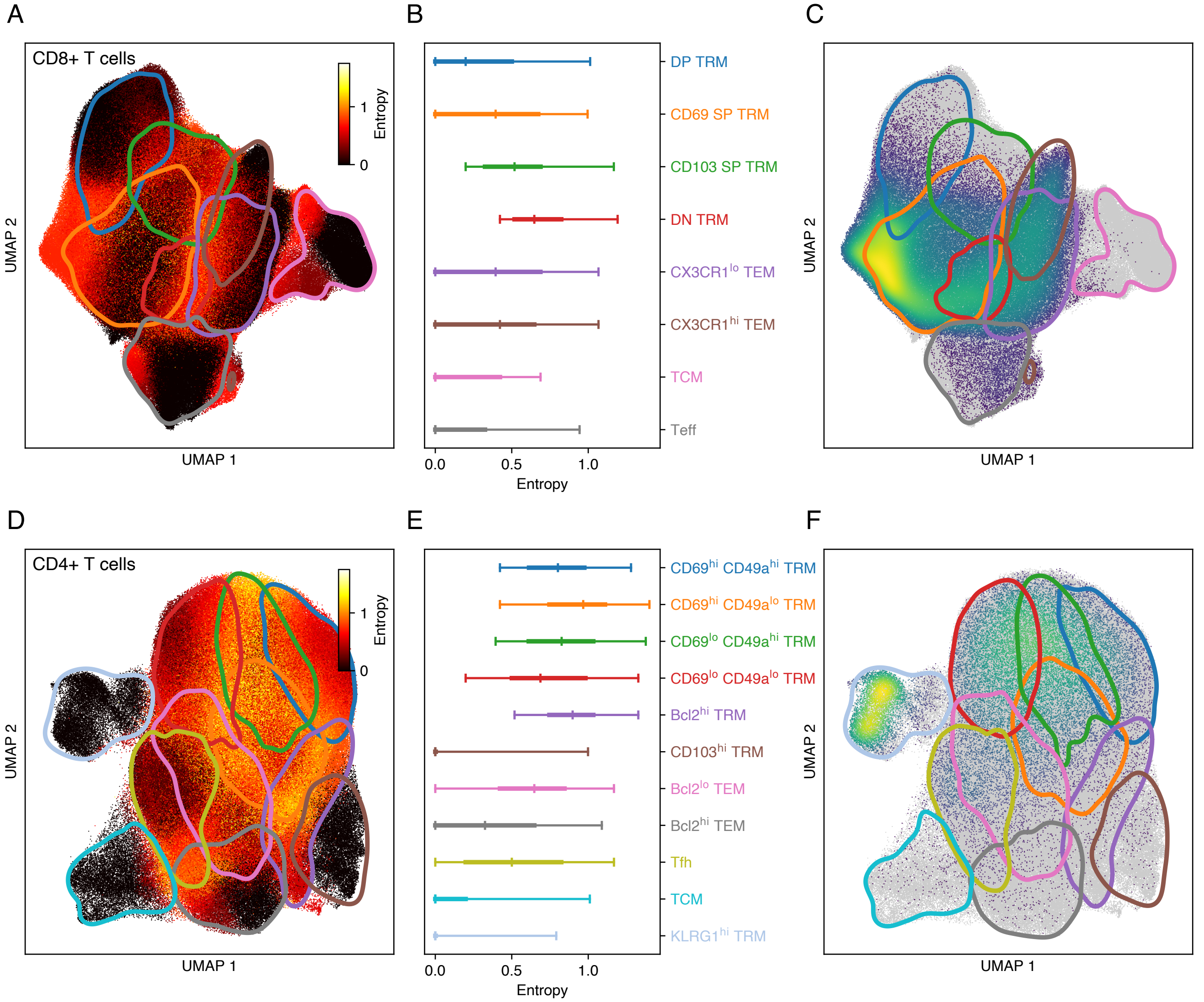

Figure S3: Validation of population assignment in the sequential approach. Results are based on data from $n = 27$ mice. **A.** Entropy of CD8 T cell population assignment based on 20 Leiden clustering runs with different random seeds. Black dots corresponds to very certain assignments, yellow dots to highly uncertain assignments. The colored contours indicate the location of the different sub-populations in the UMAP. **B.** Entropy distribution per cluster. The bar plots show the median, IQR and 2.5 - 97.5 percentile range. The color of the bars and labels correspond to the contours in panel A. **C.** Distribution of IAV NP-specific CD8 T cells in the UMAP with contours indicating clusters. Gray dots indicate bulk antigen-experienced cells. **D-F.** Same as panels A-C, but for CD4 T cell data.

bioRxiv preprint doi: https://doi.org/10.1101/2024.07.08.602409; this version posted February 25, 2025. The copyright holder for this preprint (which was not certified by peer review) is the author/funder, who has granted bioRxiv a license to display the preprint in perpetuity. It is made available under aCC-BY 4.0 International license.

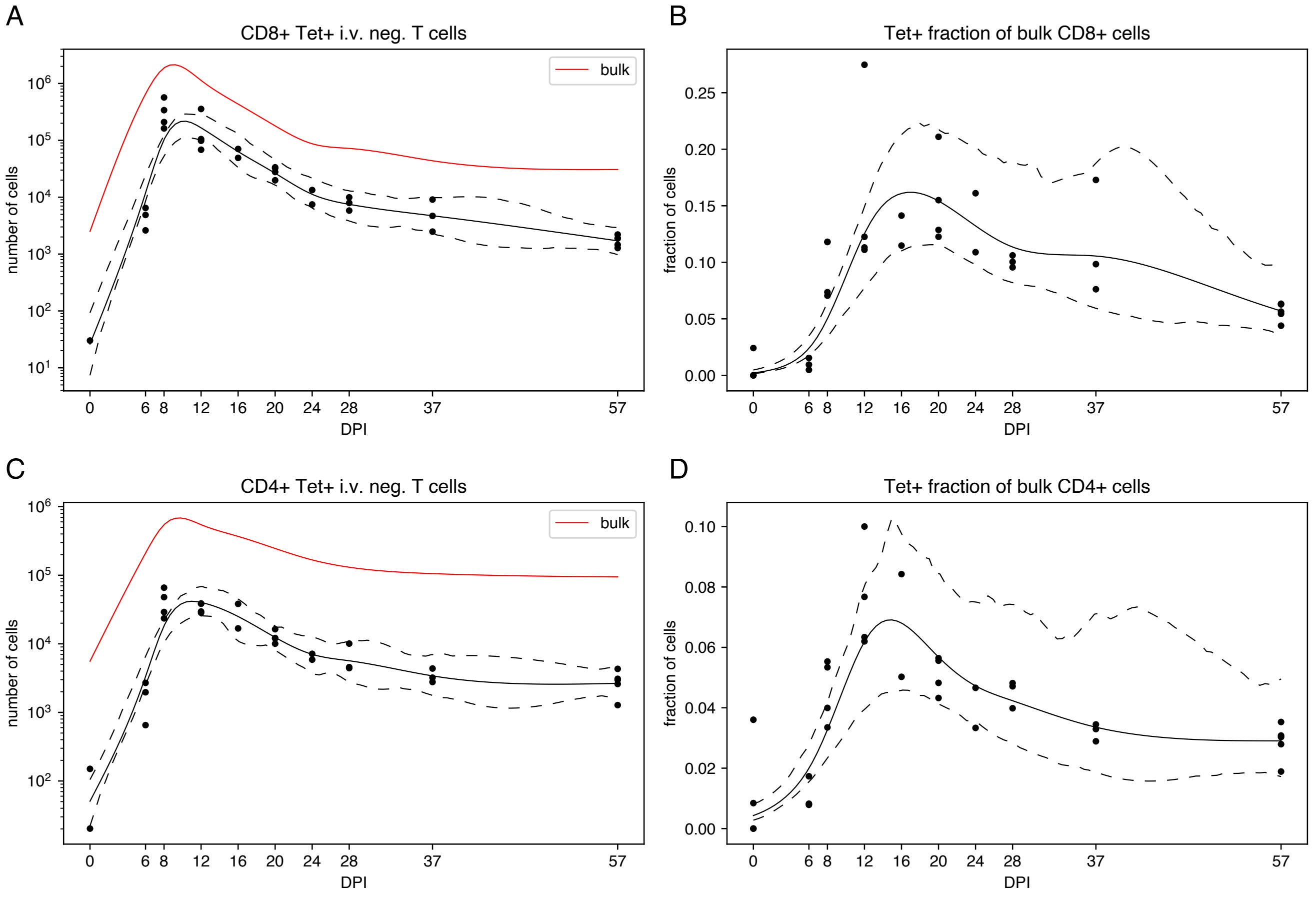

Figure S4: Timecourses of NP-specific T cells. Results are based on data from $n = 34$ mice. **A.** Number of NP-specific CD8 T cells, as a function of post-infection sampling time. The curve represents a spline fit to the log-transformed T cell counts, and the dashed lines represent the 95% confidence envelope (estimated by bootstrapping residuals). The red curve indicates the number of polyclonal CD8 T cells (cf. Fig. 1B). **B.** The Tet$^+$ fraction of bulk CD8 T cells in the lung niche. Splines are fitted on the logit scale. **C.** Number of NP-specific CD4 T cells. **D.** The Tet$^+$ fraction of bulk CD4 T cells.

bioRxiv preprint doi: https://doi.org/10.1101/2024.07.08.602409; this version posted February 25, 2025. The copyright holder for this preprint (which was not certified by peer review) is the author/funder, who has granted bioRxiv a license to display the preprint in perpetuity. It is made available under aCC-BY 4.0 International license.

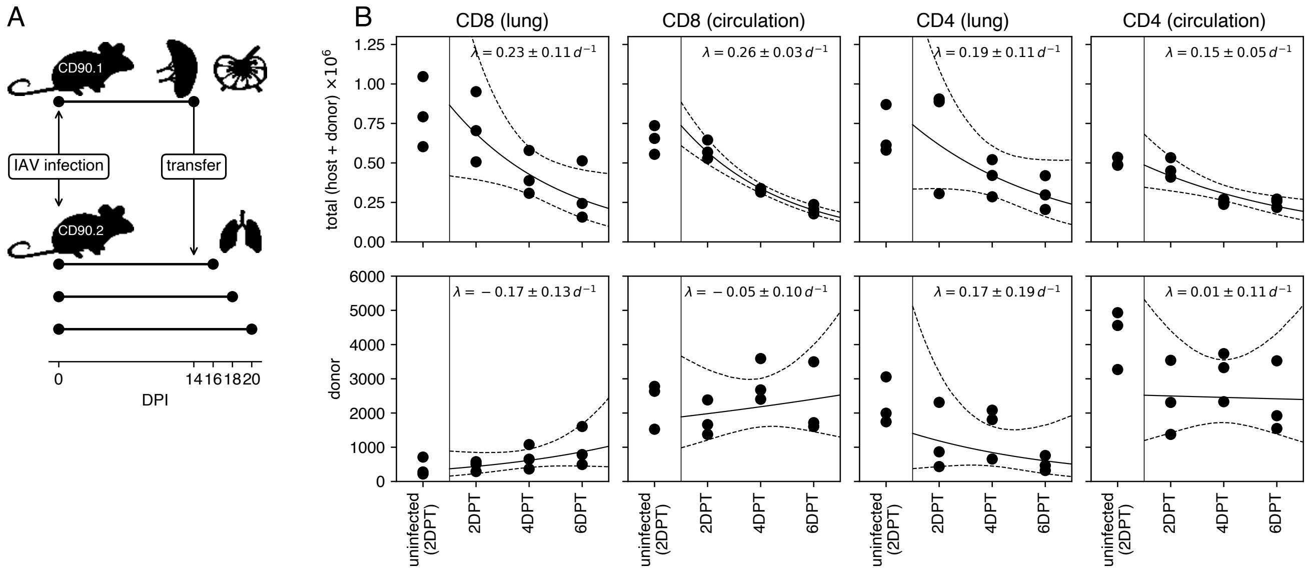

Figure S5: Congenic transfer experiment to assess the amount of ingress during the memory phase. Results are based on data from $n = 12$ mice in total, with 3 mice per group. **A.** Design of the congenic transfer experiment. **B.** Numbers of protected and labeled, antigen-experienced CD8 and CD4 T cells from host and donors combined (upper panels) and donors only (lower panels). We fitted a log-linear model to the cell counts (solid line: ML estimate, dashed lines: 95% confidence envelope). The indicated λ the estimated net loss rate ($\pm$ standard error).

bioRxiv preprint doi: https://doi.org/10.1101/2024.07.08.602409; this version posted February 25, 2025. The copyright holder for this preprint (which was not certified by peer review) is the author/funder, who has granted bioRxiv a license to display the preprint in perpetuity. It is made available under aCC-BY 4.0 International license.

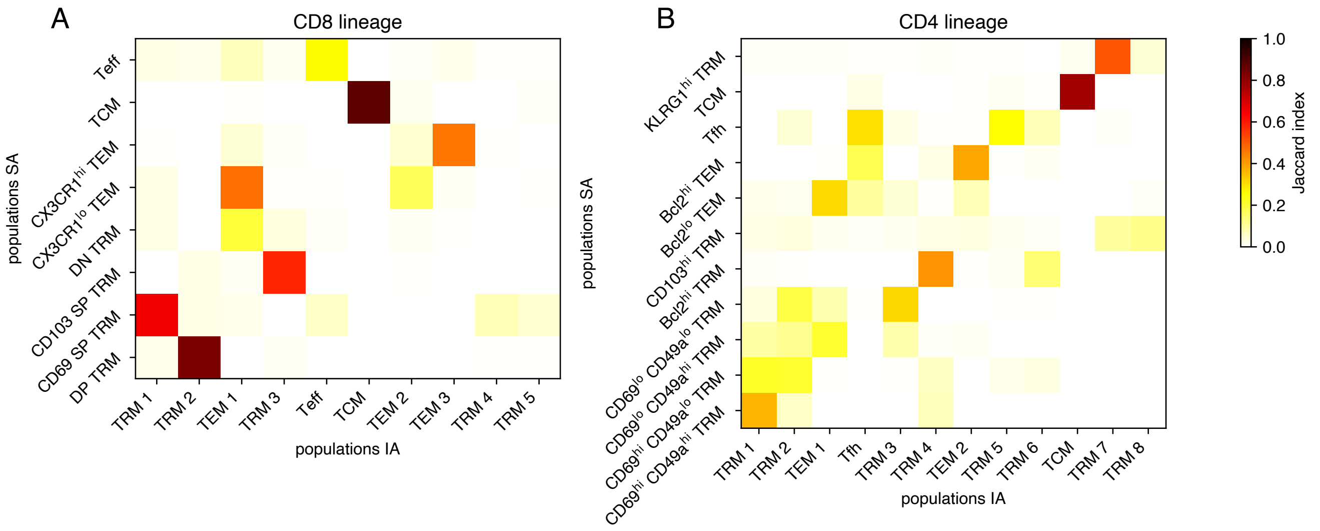

Figure S6: Comparison of results from the integrated and sequential approaches. Results are based on data from $n = 27$ mice. The sequential (SA) and integrated (IA) approaches both assign each cell to a subpopulation, but for any given cell this assignment may differ between the approaches. To quantify the similarity, we calculated the Jaccard index for each pair of sub-populations (one from the IA and the other from the SA). The Jaccard index is ratio of the number of cells that are in both clusters, and the number of cells that are in either one of the clusters. **A.** Results for CD8 T cell data. **B.** Results for CD4 T cell data.

bioRxiv preprint doi: https://doi.org/10.1101/2024.07.08.602409; this version posted February 25, 2025. The copyright holder for this preprint (which was not certified by peer review) is the author/funder, who has granted bioRxiv a license to display the preprint in perpetuity. It is made available under aCC-BY 4.0 International license.

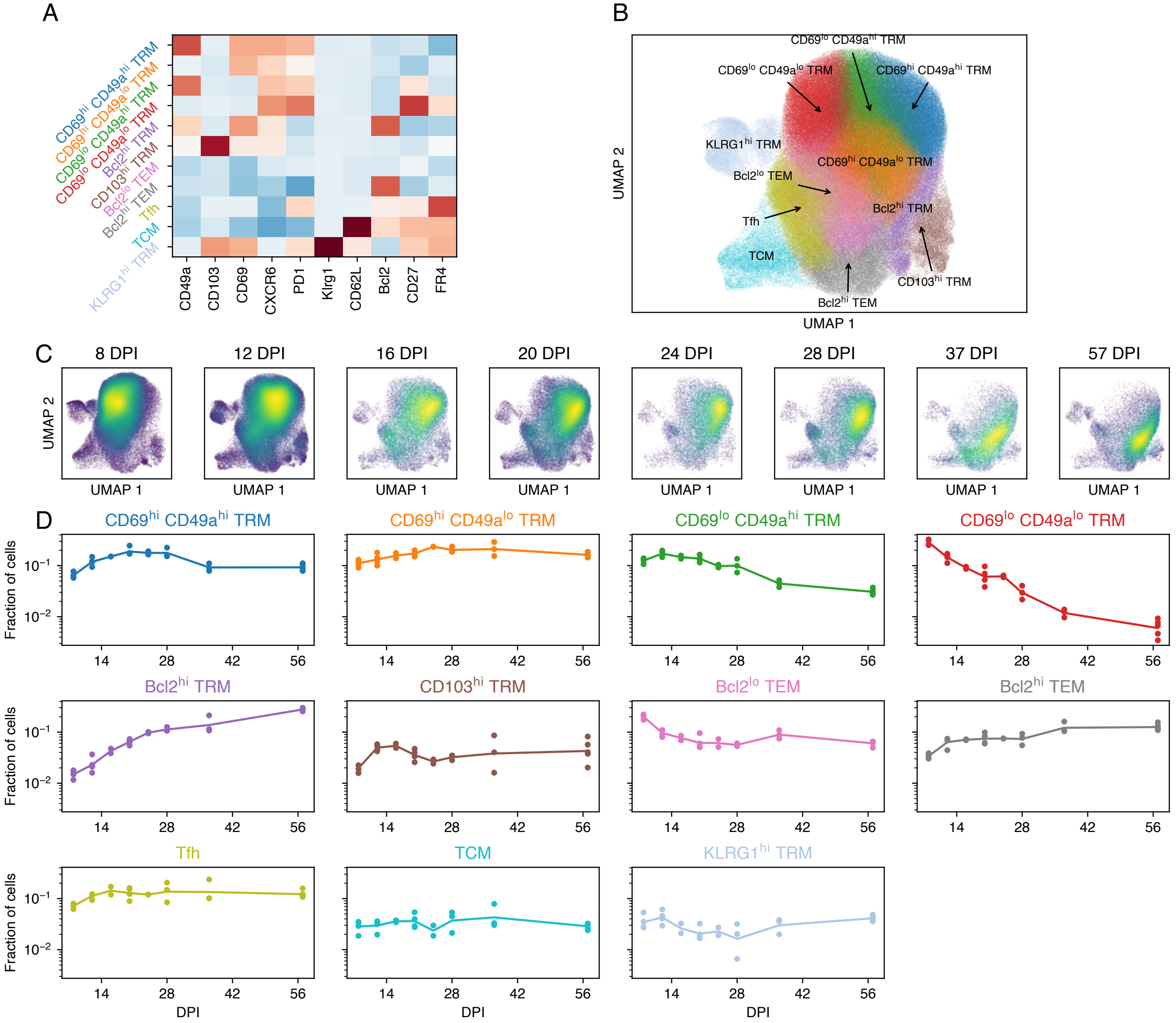

Figure S7: Pre-processing CD4$^+$ T cell flow cytometry data for the sequential approach. Results are based on data from $n = 27$ mice. **A.** Marker expression heatmap for selected markers and consensus T-cell populations. **B.** UMAP of the marker expression data, colored by annotation. **C.** UMAPs of marker expression data, split by day post infection (DPI). The color scale reflects cell density in UMAP space. **D.** Time series of the fraction of cells in each cluster. The lines show a linear interpolation on the log scale.

bioRxiv preprint doi: https://doi.org/10.1101/2024.07.08.602409; this version posted February 25, 2025. The copyright holder for this preprint (which was not certified by peer review) is the author/funder, who has granted bioRxiv a license to display the preprint in perpetuity. It is made available under aCC-BY 4.0 International license.

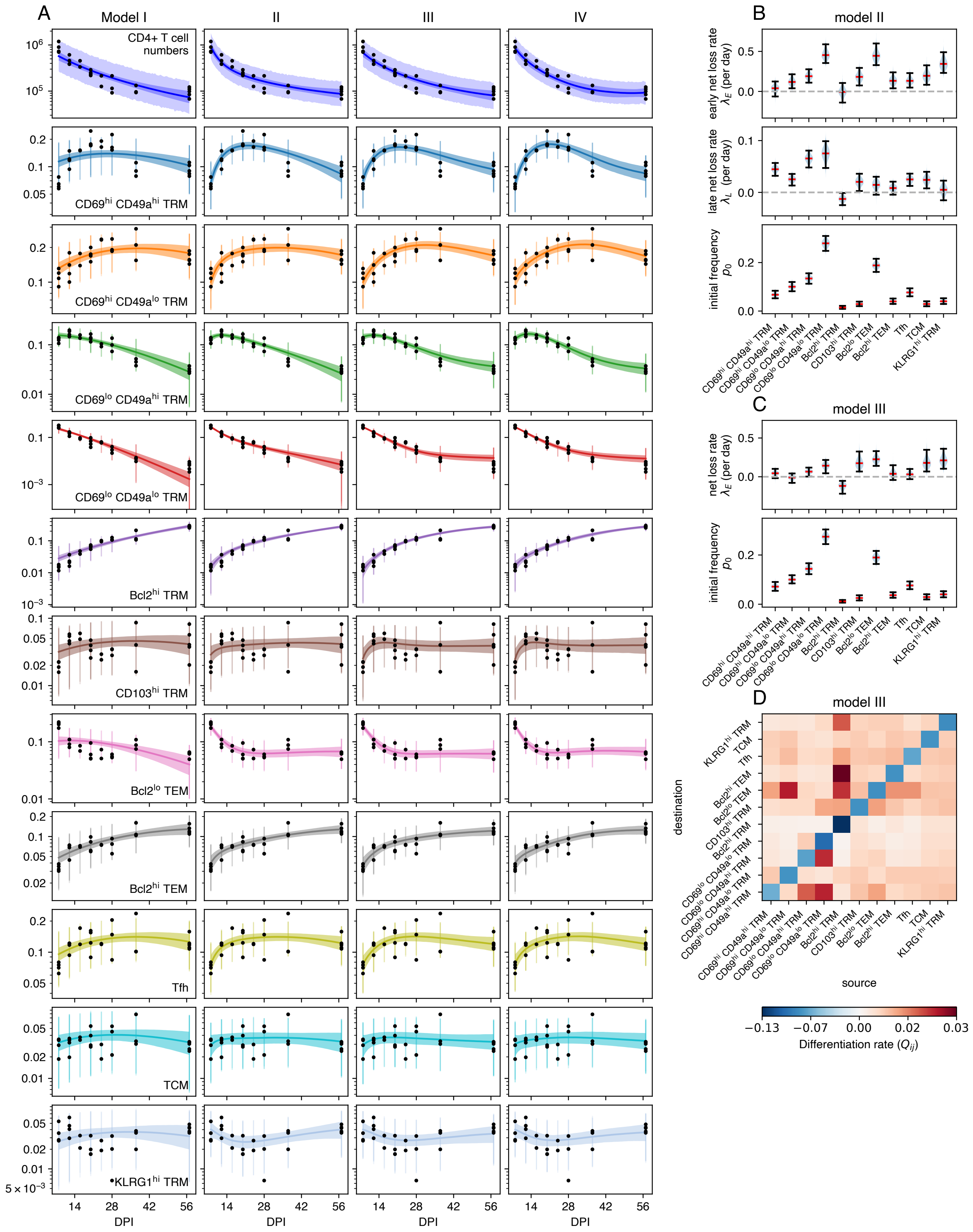

Figure S8: Model fits to CD4 T cell timeseries using the sequential approach. Results are based on data from $n = 27$ mice. **A.** Data and predictions from fitted models. Top panels show total antigen-experienced CD4 T cell counts in the lung, other panels show relative population sizes of each of the sub-populations. **B.** Parameter estimates using model II. **C.** Parameter estimates using model III. **D.** Estimated differentiation matrix in model III.

bioRxiv preprint doi: https://doi.org/10.1101/2024.07.08.602409; this version posted February 25, 2025. The copyright holder for this preprint (which was not certified by peer review) is the author/funder, who has granted bioRxiv a license to display the preprint in perpetuity. It is made available under aCC-BY 4.0 International license.

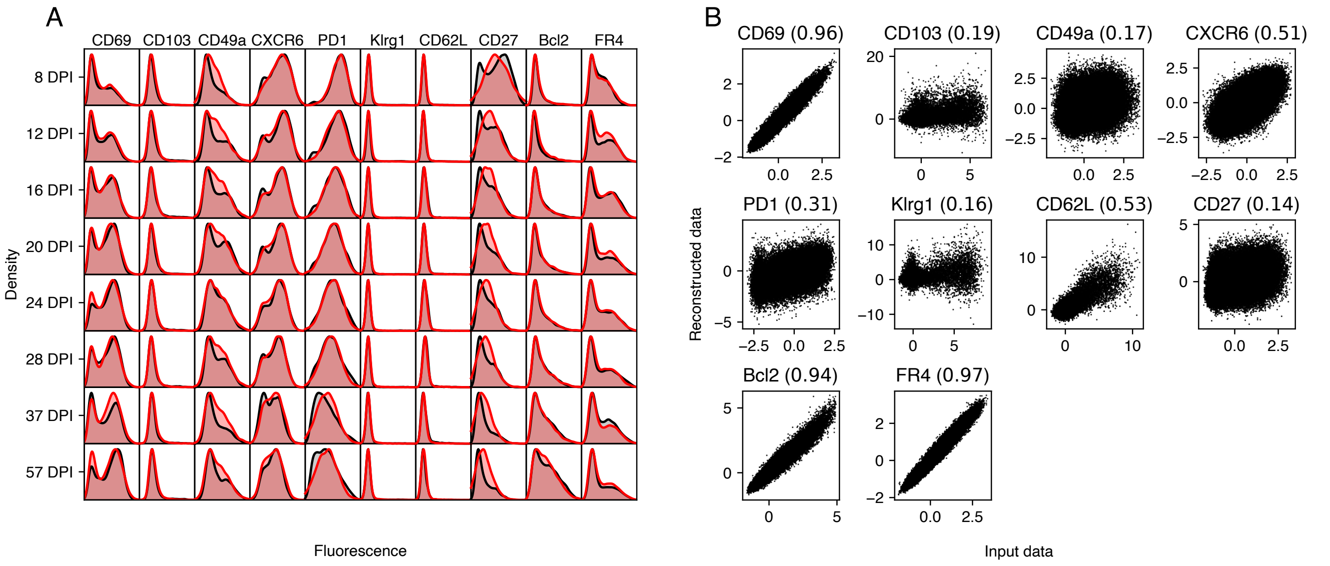

Figure S9: Posterior predictive checks for the integrated approach with CD4 T cell data. Results are based on data from $n = 27$ mice. **A.** Marginal distributions of marker expression (cf. Fig. 1, panel I). Data is shown in black, simulated data is shown in red. **B.** Input data and reconstruction using the auto-encoder model. The number in brackets is the coefficient of determination (R^2).

bioRxiv preprint doi: https://doi.org/10.1101/2024.07.08.602409; this version posted February 25, 2025. The copyright holder for this preprint (which was not certified by peer review) is the author/funder, who has granted bioRxiv a license to display the preprint in perpetuity. It is made available under aCC-BY 4.0 International license.

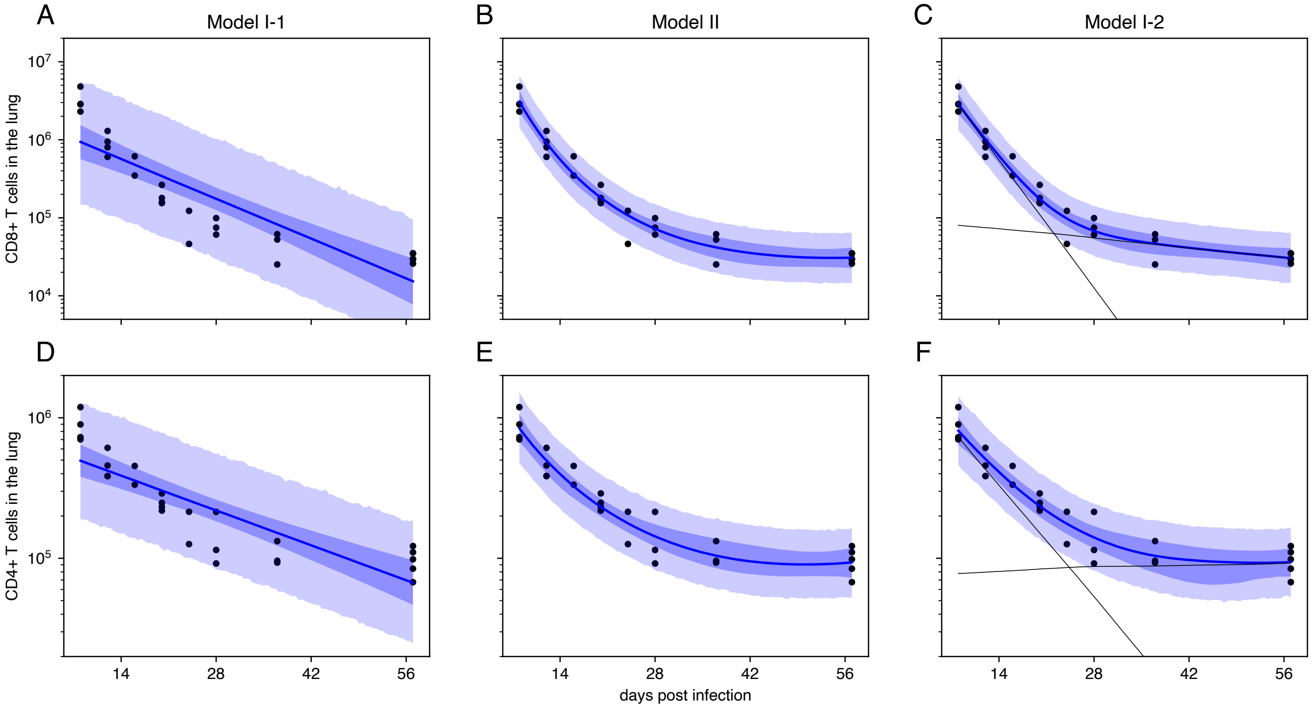

Figure S10: Model fits to cell count data alone. Results are based on data from $n = 27$ mice. **A.** time-homogeneous model with a single compartment (i.e. a log-linear model) fit to CD8 T-cell count data (dots). The model fit is shown as a blue curve (posterior median), with 95% CrI as a dark-blue band. The light-blue band shows the posterior predictive interval (i.e. simulated observations). **B.** Fit of model with a single compartment, but with time-dependent net loss rates $\lambda(t)$. **C.** Fit of time-homogeneous model with two compartments. The population sizes (posterior median) of the two populations are shown as black curves. **D-F.** Fits of the three model to CD4 T cell counts.

bioRxiv preprint doi: https://doi.org/10.1101/2024.07.08.602409; this version posted February 25, 2025. The copyright holder for this preprint (which was not certified by peer review) is the author/funder, who has granted bioRxiv a license to display the preprint in perpetuity. It is made available under aCC-BY 4.0 International license.

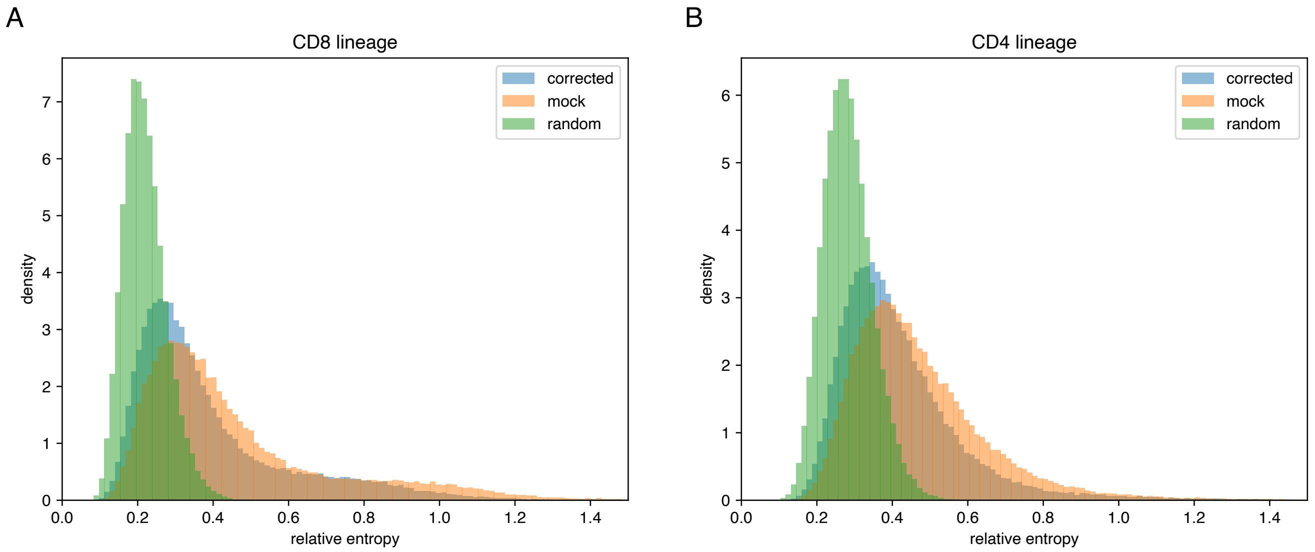

Figure S11: The effect of batch correction in the integrated approach. Results are based on data from $n = 27$ mice. Shown is the entropy of the experimental batch distribution around each cell, using the latent vector z and its nearest neighbors. Values for batch-corrected latent vectors are shown in blue. Mock corrected values are shown in orange, and values for randomized batch information are shown in green. Panels A and B show CD8 and CD4 data, respectively. The distributions are capped at 1.5 as there was a very small number of cells with high relative entropy.

bioRxiv preprint doi: https://doi.org/10.1101/2024.07.08.602409; this version posted February 25, 2025. The copyright holder for this preprint (which was not certified by peer review) is the author/funder, who has granted bioRxiv a license to display the preprint in perpetuity. It is made available under aCC-BY 4.0 International license.

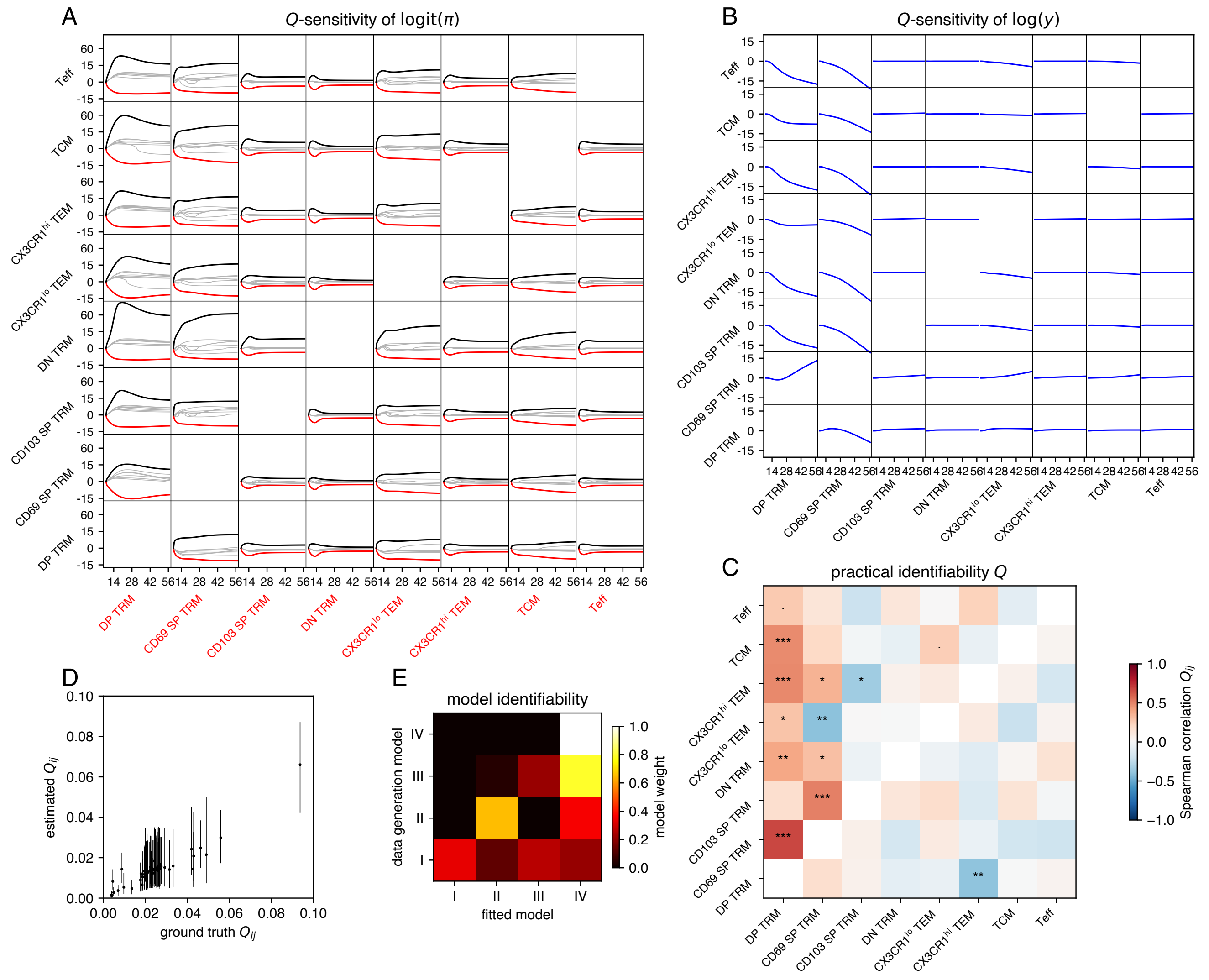

Figure S12: Identifiability and sensitivity analysis for the CD8 T cell lineage. A. Sensitivity of relative cluster sizes $(\pi_k(t))$ with respect to differentiation rates Q_{ij}. Each plot contains $d = 8$ curves $\partial \text{logit}(\pi_k(t))/\partial Q_{ij}$. The primary effects are highlighted in black $(i = k)$ and red $(j = k)$, while the secondary effects are shown in gray. The y-axes are shown on a square-root-scale. **B.** Sensitivity of total population size $(Y(t))$ with respect to differentiation rates Q_{ij}. The curves correspond to $\partial \log(Y(t))/\partial Q_{ij}$. **C.** Practical identifiability scores of differentiation rates Q_{ij}. The heatmap shows the correlation coefficient between the ground truth value of Q_{ij}, and the estimated value. The stars indicate the levels of statistical significance ($\cdot\, p < 0.1$, $*\, p < 0.05$, $**\, p < 0.01$, $*{*}*\, p < 0.001$) based on a range of 51 ground truth Q_{ij} values. **D.** A single pseudo-data set is simulated with model IV, using parameters estimated from the true data. Model IV is then fit to the pseudo-data, and for each pair of populations (i, j) we show the estimate versus the ground truth value of Q_{ij}. **E.** Model identifiability. Data was simulated with and fit to each of the four models, resulting in 16 model fits. For a each simulated dataset, the four model fits are compared with model weights (shown in color). High diagonal model weights indicate that the ground-truth model is correctly identified. Shown is the median of 3 simulations for each model.

bioRxiv preprint doi: https://doi.org/10.1101/2024.07.08.602409; this version posted February 25, 2025. The copyright holder for this preprint (which was not certified by peer review) is the author/funder, who has granted bioRxiv a license to display the preprint in perpetuity. It is made available under aCC-BY 4.0 International license.

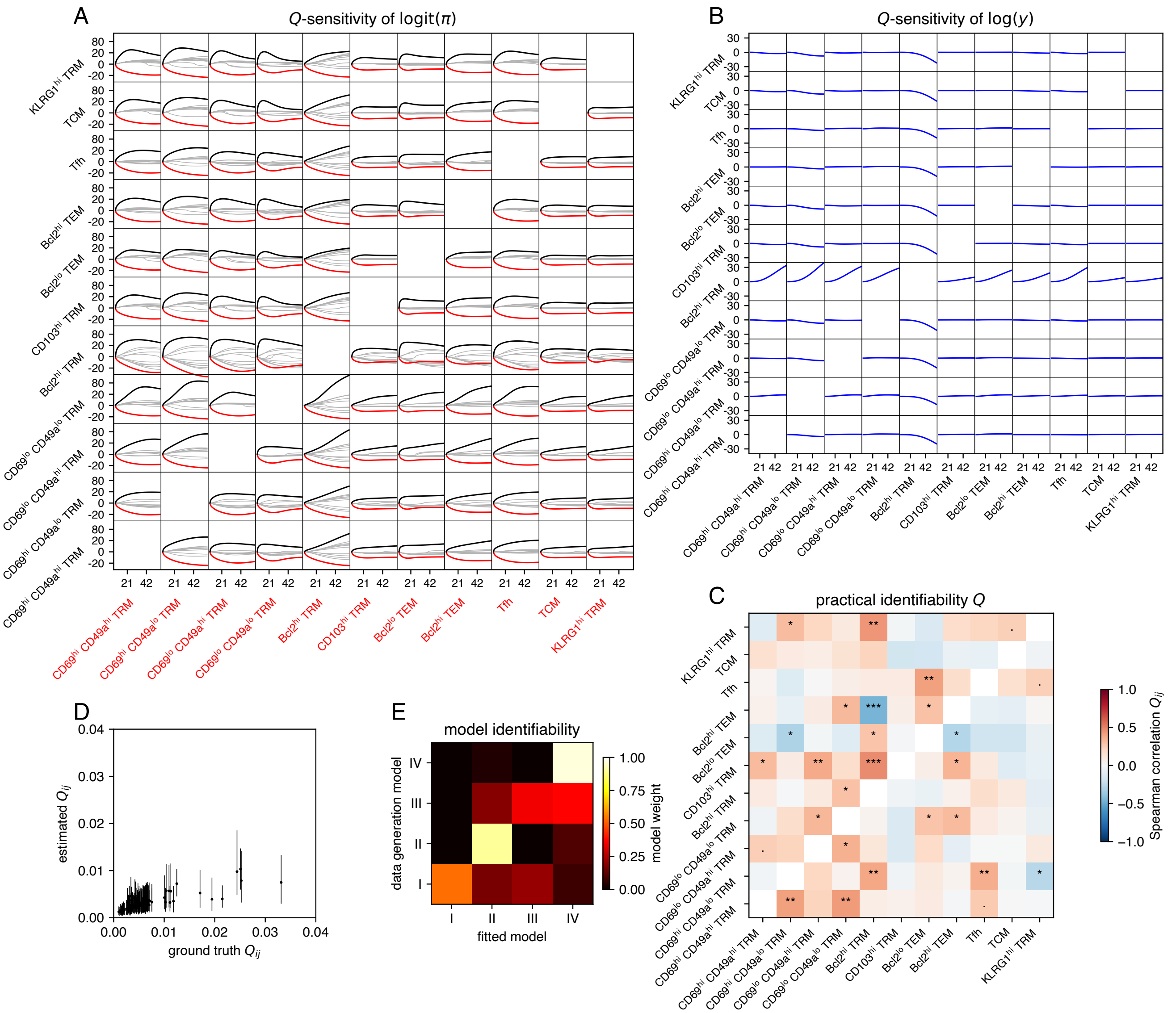

Figure S13: Identifiability and sensitivity analysis for the CD4 T cell lineage. See the caption of Fig. S12 for details. In this case we have $d = 11$ populations, and for panel D we simulated and fitted with model III.

bioRxiv preprint doi: https://doi.org/10.1101/2024.07.08.602409; this version posted February 25, 2025. The copyright holder for this preprint (which was not certified by peer review) is the author/funder, who has granted bioRxiv a license to display the preprint in perpetuity. It is made available under aCC-BY 4.0 International license.

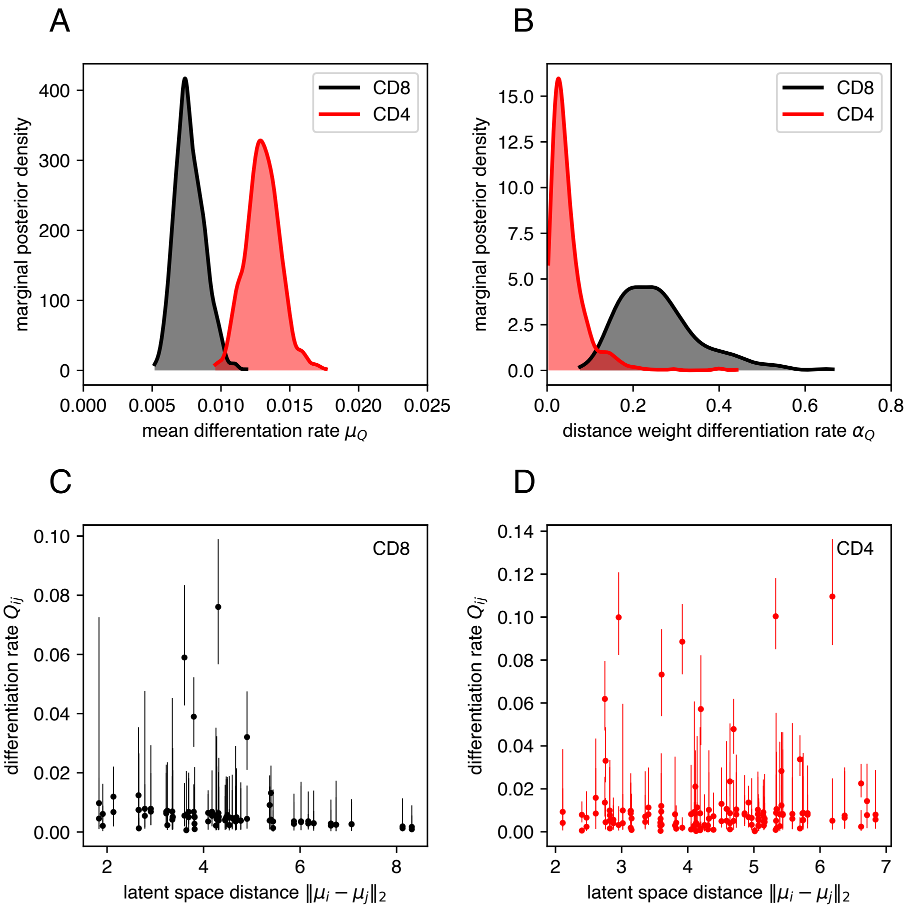

Figure S14: Cluster similarity informed prior distribution on the Q-matrix. A. Marginal posterior density of the mean differentiation rate μ_Q. **B.** Marginal posterior density of the weight α_Q of the distance matrix $D_{ij} = \|\mu_i - \mu_j\|_2$ on the differentiation matrix elements Q_{ij}. **C.** and **D.** 95% credible intervals (lines) and posterior medians (dots) of Q_{ij} as a function of the distance between the mixture components i and j in the latent space.

bioRxiv preprint doi: https://doi.org/10.1101/2024.07.08.602409; this version posted February 25, 2025. The copyright holder for this preprint (which was not certified by peer review) is the author/funder, who has granted bioRxiv a license to display the preprint in perpetuity. It is made available under aCC-BY 4.0 International license.

SI Text A Ingress contributes minimally to the population dynamics of lung T_{RM} during the memory phase

To inform the construction of models of cell dynamics within the lung, we aimed to quantify the extent to which they were supplemented by new immigrants following the peak of infection. Our approach is illustrated in Fig. S5A. To do this we simultaneously infected cohorts of CD90.1 and CD90.2 congenic mice with IAV, and at day 14 post infection, transferred 10^6 cells from the spleens and mediastinal lymph nodes of the CD90.1 (donor) mice to the CD90.2 (host) mice (see Methods). At 16, 18 and 20 days post infection (2, 4, and 6 days post transfer, respectively), lung tissue was collected from the host mice, and analyzed with flow cytometry. The kinetics of accumulation of any donor cells in the host lung tissue would then reflect the degree of ongoing recruitment of new T_{RM} from circulating influenza-specific memory T cells.

Between 16 and 20 days post infection, the total numbers of CD4 and CD8 T cells (host+donor) fell by roughly 50% (Fig. S5B), consistent with the rates of decline observed in Fig. 1B-C. During the same time window, donor-derived cells were detectable in the lung but at low numbers that represented between 0.05-0.01% of transferred cells, and remained stable in number between 2-6 days post transfer. Between 16-18 DPI the rate of increase was 0.2 per day (95% CI $[-0.1, 0.4]$) for $CD8^+$ T cells and -0.2 per day for $CD4^+$ T cells (95% CI $[-0.5, 0.2]$). The number of donor cells in circulation (i.v. labeled cells from the lung sample) were consistently higher than cells in the tissue (CD8: 4.7 fold, $p = 2 \times 10^{-4}$; CD4: 3 fold, $p = 4 \times 10^{-3}$ paired t-test), while the total number of cells in circulation and in tissue were comparable (CD8: $p = 0.13$, CD4: $p = 0.1$). The number of donor cells in the lung tissue from uninfected donors was comparable to the number of cells from infected donors, indicating that the rate of ingress does not depend on IAV-specificity. We conclude that ingress of antigen-experienced T cells into the lung occurs at very low levels from at least 14 DPI onward.

bioRxiv preprint doi: https://doi.org/10.1101/2024.07.08.602409; this version posted February 25, 2025. The copyright holder for this preprint (which was not certified by peer review) is the author/funder, who has granted bioRxiv a license to display the preprint in perpetuity. It is made available under aCC-BY 4.0 International license.

SI Text B A two-compartment, time-homogeneous model can explain the time-course of CD8 T cell numbers

At first glance, the CD8 and CD4 T cell count data resemble typical biphasic exponential decay patterns [61]. Such time series can easily be modeled using two exponentially declining populations that decrease at two different rates. Initially, the more rapidly declining population is more abundant, but soon it is replaced (relatively) by the population that declines at a slower rate.

Because of this resemblance, it might be surprising that we need time-dependent loss rates or differentiation to model the T-cell populations in the lung. To make this initial expectation more rigorous, we fit three different models (denoted I-1, II and I-2) to the CD8 and CD4 T-cell count data alone. Model I-1 is time-homogeneous with only a single population (Fig S10A and D). This model can not describe the biphasic decay pattern seen in the data, and requires a large standard deviation for the error model (CD8: $\sigma_M = 0.86$, 95% CrI $[0.67, 1.17]$; CD4: $\sigma_M = 0.45$, 95% CrI $[0.35, 0.61]$).

Model II again has a single population, but now we include a time-dependent net loss rate $\lambda(t)$ as in Eqn. 2. This model fits the T-cell count data very well (Fig S10B and E, and requires a much smaller standard deviation for the error model (CD8: $\sigma_M = 0.34$, 95% CrI $[0.26, 0.46]$; CD4: $\sigma_M = 0.25$, 95% CrI $[0.19, 0.36]$). Finally, model I-2 has constant decay rates, but assumes that there are two distinct T-cell populations that decay at different rates. Judging from the posterior predictive check (Fig. S10C and E), this model fits the data as well as model II, and requires similar σ_M values as model II.

In terms of LOO-IC, model II and I-2 are indistinguishable (CD8: ΔLOO-IC $= 0.3 \pm 1.5$; CD4: ΔLOO-IC $= 0.3 \pm 0.5$), while model I-1 is significantly worse (CD8: ΔLOO-IC $= 24 \pm 3$; CD4: ΔLOO-IC $= 15 \pm 3$). This means that model II and I-2 describe the count data equally well, and hence using count data alone, it is not possible to distinguish between a model with two populations or a time-dependent net loss rate.

bioRxiv preprint doi: https://doi.org/10.1101/2024.07.08.602409; this version posted February 25, 2025. The copyright holder for this preprint (which was not certified by peer review) is the author/funder, who has granted bioRxiv a license to display the preprint in perpetuity. It is made available under aCC-BY 4.0 International license.

SI Text C The geometry of time-homogeneous loss of independent populations

The simplest model we considered has a geometric property that allows it to be easily tested against data. Suppose that the populations of T cells are independent (i.e. no differentiation, or $Q = 0$) and that their net loss rates are constant (i.e. time homogeneous). The model is then

$$\frac{dX}{dt} = -\lambda \circ X \tag{1}$$

with initial condition $X(t_0) = X_0$. This model admits the following solution

$$X(t) = X_0 \circ \exp(-\lambda(t - t_0)), \tag{2}$$

where the exponential function is taken element-wise. Again, we write $Y(t) = \sum_{i=1}^{d} X_i(t)$ for the total population size and $\pi_i(t) = X_i(t)/Y(t)$ for the population fractions.

When we look at the trajectories $\pi_i(t)$ on a logarithmic scale, a striking property is that they are all concave (Fig 3B and Fig. S8A, first column). We can see this mathematically as follows. A twice differentiable function is concave if the second derivative is non-positive. As the log of $\pi_i(t)$ is given by

$$\log \pi_i(t) = \log(X_{0,i}) - \lambda_i(t - t_0) - \log Y(t)\,, \tag{3}$$

the second derivative is given by

$$\frac{d^2}{dt^2} \log \pi_i(t) = -\frac{d^2}{dt^2} \log Y(t), \tag{4}$$

which does not depend on the population index i. The second derivative of $\log Y(t)$ is given by

$$\frac{d^2}{dt^2} \log Y(t) = \frac{Y(t)Y''(t) - Y'(t)^2}{Y(t)^2} \tag{5}$$

so its sign is determined by $Y(t)Y''(t) - Y'(t)^2$. We have $Y'(t) = -\sum_{i=1}^{d} \lambda_i X_i(t)$, and $Y''(t) = \sum_{i=1} \lambda_i^2 X_i(t)$. Now consider the vectors a and b given by $a_i = -\lambda_i \sqrt{X_i}$ and $b_i = \sqrt{X_i}$. By the Cauchy-Schwartz inequality, we have $\langle a, b\rangle^2 \leq \langle a, a\rangle \langle b, b\rangle$, and hence

$$Y'(t)^2 = \left(-\sum_{i=1}^{d} \lambda_i X_i\right)^2 \leq \sum_{i=1}^{d} \lambda_i^2 X_i(t) \sum_{i=1}^{d} X_i = Y''(t)Y(t) \tag{6}$$

which means that $\frac{d^2}{dt^2} \log Y(t) \geq 0$. Hence $\log \pi_i(t)$ is concave.

bioRxiv preprint doi: https://doi.org/10.1101/2024.07.08.602409; this version posted February 25, 2025. The copyright holder for this preprint (which was not certified by peer review) is the author/funder, who has granted bioRxiv a license to display the preprint in perpetuity. It is made available under aCC-BY 4.0 International license.

SI Text D Batch correction

To measure how well batch correction performed in our integrated approach, we wanted to quantify the extent to which cells from different mice were well-mixed in phenotypic space [32]. To do this, we computed the K-nearest-neighbor graph of the latent representation z_i of the cells using the `scikit-learn` package [62]. For each cell i, we then counted the number of neighbors $k_{i,s}$ derived from mouse s (including the focal cell). If cells are well mixed, all of these counts should be distributed according to the sample sizes n_s of the animals s. A convenient measure of how well the distributions of the $k_{i,s}$ match is the relative entropy, given by

$$H_i = -\sum_s n_s/N \left(\log(k_{i,s}/K) - \log(n_s/N)\right), \tag{7}$$

where $N = \sum_s n_s$ is the total number of cells from all animals. A lower relative entropy corresponds to more homogeneous mixing.

Fig. S11 shows the distribution of H_i as a blue histogram for the CD8 (panel A) and CD4 data (panel B). These distributions are difficult to interpret in isolation and so we compared them with two extremes. First, we computed the distribution of H_i under the assumption that all cells from all animals were homogeneously distributed. In this case we would have $\vec{k}_i \sim \mathrm{Multinomial}(K, \vec{n}/N)$. We randomly generated samples for each cell i and the resulting distribution is shown as an green histogram in Fig. S11. The other extreme is the case when we switch off the effect of batch-correction. To accomplish this, we picked the animal s^* with the largest sample size n_{s^*} as a reference. We then used the encoder network to compute latent representations z_i from the pairs (x_i, s^*), instead of the usual pairs (x_i, s_i). This means that we simulate the scenario in which all cells came from the same animal s^*. The resulting distribution of H_i is shown as an orange histogram in Fig. S11.

Our relative entropy-based analysis shows that batch correction is effective at aligning the distributions of cells from different animals within the latent space. However, the relative entropy remains much higher than expected under a perfectly homogeneous distribution (green vs. orange histogram in Fig. S11). This is due to the fact that mice are sampled at different DPI, and the phenotype distribution is dependent on time, and possibly because all data was collected at the same time (Fig. 1), reducing batch effects.

bioRxiv preprint doi: https://doi.org/10.1101/2024.07.08.602409; this version posted February 25, 2025. The copyright holder for this preprint (which was not certified by peer review) is the author/funder, who has granted bioRxiv a license to display the preprint in perpetuity. It is made available under aCC-BY 4.0 International license.

SI Text E Sensitivity Analysis

To evaluate the sensitivity of ODE model (1) to the parameters, we derived and numerically integrated the sensitivity equations corresponding to the system. We therefore have to calculate the derivative of the state at each time point with respect to the parameter of interest. We can do this by interchanging the time derivative and the parameter gradient operator. Recall that the model is given by

$$\frac{d}{dt}X = -\lambda(t) \circ X + QX\,, \quad \text{where} \quad \lambda(t) = (\lambda_E - \lambda_L)e^{-u(t-t_0)} + \lambda_L \tag{8}$$

For completeness, we derive sensitivity equations for all parameters, altough we are mainly focussing on Q in the main text. The sensitivity equations for parameter u are then derived as follows.

$$\begin{aligned}\frac{d}{dt}\frac{\partial X}{\partial u} &= \frac{\partial}{\partial u}\big(-\lambda(t) \circ X + QX\big)\\ &= -\frac{\partial \lambda(t)}{\partial u}X - \lambda(t)\frac{\partial X}{\partial u} + Q\frac{\partial X}{\partial u}\\ &= (t-t_0)e^{-u(t-t_0)}(\lambda_L - \lambda_E) \circ X - \lambda(t)\frac{\partial X}{\partial u} + Q\frac{\partial X}{\partial u}\end{aligned} \tag{9}$$

Next, we derive equations for the net loss rates. If we interpret $\partial/\partial\lambda_E$ as a row vector, $\partial X/\partial\lambda_E$ is a $d \times d$ matrix.

$$\begin{aligned}\frac{d}{dt}\frac{\partial X}{\partial \lambda_E} &= \frac{\partial}{\partial \lambda_E}\big(-\lambda(t) \circ X + QX\big)\\ &= -\text{diag}(X)\frac{\partial \lambda(t)}{\partial \lambda_E} - \text{diag}(\lambda(t))\frac{\partial X}{\partial \lambda_E} + Q\frac{\partial X}{\partial \lambda_E}\\ &= -e^{-u(t-t_0)}\text{diag}(X) - \text{diag}(\lambda(t))\frac{\partial X}{\partial \lambda_E} + Q\frac{\partial X}{\partial \lambda_E}\end{aligned} \tag{10}$$

Likewise, we get for $\partial X/\partial\lambda_L$

$$\frac{d}{dt}\frac{\partial X}{\partial \lambda_L} = -(1 - e^{-u(t-t_0)})\text{diag}(X) - \text{diag}(\lambda(t))\frac{\partial X}{\partial \lambda_L} + Q\frac{\partial X}{\partial \lambda_L} \tag{11}$$

To derive sensitivity equations for the generator matrix Q, we first derive them for a general matrix A and the system $\frac{d}{dt}X = -\lambda(t) \circ X + AX$, and then impose restrictions on the diagonal elements.

$$\frac{d}{dt}\frac{\partial X_k}{\partial A_{ij}} = -\lambda_k(t)\frac{\partial X_k}{\partial A_{ij}} + \sum_{\ell=1}^{d} A_{k\ell}\frac{\partial X_\ell}{\partial A_{ij}} + \delta_{ik}X_j \tag{12}$$

We now substitute $A_{ij} = Q_{ij}$ such that $A_{jj} = -\sum_{i=1}^{d} Q_{ij}$ and get for $i \neq j$

$$\frac{\partial X_k}{\partial Q_{ij}} = \frac{\partial X_k}{\partial A_{ij}} - \frac{\partial X_k}{\partial A_{jj}} \tag{13}$$

bioRxiv preprint doi: https://doi.org/10.1101/2024.07.08.602409; this version posted February 25, 2025. The copyright holder for this preprint (which was not certified by peer review) is the author/funder, who has granted bioRxiv a license to display the preprint in perpetuity. It is made available under aCC-BY 4.0 International license.

Therefore we get for $i \neq j$

$$\begin{aligned}\frac{d}{dt}\frac{\partial X_k}{\partial Q_{ij}} &= -\lambda_k(t)\frac{\partial X_k}{\partial A_{ij}} + \sum_{\ell=1}^{d} A_{k\ell}\frac{\partial X_\ell}{\partial A_{ij}} + \delta_{ik}X_j + \lambda_k(t)\frac{\partial X_k}{\partial A_{jj}} - \sum_{\ell=1}^{d} A_{k\ell}\frac{\partial X_\ell}{\partial A_{jj}} - \delta_{jk}X_j \\ &= -\lambda_k(t)\frac{\partial X_k}{\partial Q_{ij}} + \sum_{\ell=1}^{d} A_{k\ell}\frac{\partial X_\ell}{\partial Q_{ij}} + (\delta_{ik} - \delta_{jk})X_j\end{aligned} \tag{14}$$

Finally, to compute the sensitivity of some transformation $f(X)$ of the state X (e.g. $f(X) = \log(Y)$, or $f(X) = \mathrm{logit}(\pi_i)$), we simply apply the chain rule.

bioRxiv preprint doi: https://doi.org/10.1101/2024.07.08.602409; this version posted February 25, 2025. The copyright holder for this preprint (which was not certified by peer review) is the author/funder, who has granted bioRxiv a license to display the preprint in perpetuity. It is made available under aCC-BY 4.0 International license.

Supporting Tables

Model	elpd_loo	p_loo	Δelpd	se Δelpd
CD8 T-cell data				
IV	-1215.5	29.1	-	-
II	-1253.9	24.3	38.4	9.6
III	-1288.4	19.3	72.9	13.2
I	-1372.9	22.2	157.4	29.9
CD4 T-cell data				
IV	-1805.4	39.0	-	-
II	-1812.1	32.5	6.7	5.1
III	-1816.4	33.9	11.0	4.9
I	-1889.7	24.9	84.3	21.2

Table S1: LOO-IC results for the sequential approach. Results are based on data from $n = 27$ mice. The models are ranked from best (top) to worst (bottom), using the "expected log predictive density" (elpd_loo) value. The p_loo value is a measure of the complexity of the model and generally increases with the number of parameters. The Δelpd value is the difference between the elpd_loo value and that of the best model. The se Δelpd is the standard error of the Δelpd.

bioRxiv preprint doi: https://doi.org/10.1101/2024.07.08.602409; this version posted February 25, 2025. The copyright holder for this preprint (which was not certified by peer review) is the author/funder, who has granted bioRxiv a license to display the preprint in perpetuity. It is made available under aCC-BY 4.0 International license.

Reagent or Resource	Source	Identifier
**Antibodies**		
Anti-Mouse Bcl2 Alexa Fluor 488	BD Biosciences	3F11; Cat. # 568426
Anti-Mouse CD103 BUV615	BD Biosciences	2E7; Cat. # 751631
Anti-Mouse CD11a PerCP-eFluor710	Thermofisher	M17/4; Cat. # 46-0111-82
Anti-Mouse CD19 BV480	BD Biosciences	1D3; Cat. # 566107
Anti-Mouse CD27 BV605	Biolegend	LG3A10; Cat. # 124249
Anti-Mouse CD3 BUV395	BD Biosciences	17A2; Cat. # 740268
Anti-Mouse CD4 APC-Cy/7	Biolegend	GK1.5; Cat. # 100412
Anti-Mouse CD44 BV786	Biolegend	IM7; Cat. # 103059
Anti-Mouse CD49a BUV737	BD Biosciences	Ha31/8; Cat. # 741776
Anti-Mouse CD62L BV570	Biolegend	MEL-14; Cat. # 104433
Anti-Mouse CD69 PE-Cy5	Biolegend	H1.2F3; Cat. # 104510
Anti-Mouse CD8 BUV805	BD Biosciences	53-6.7; Cat. # 612898
Anti-Mouse CX3CR1 APC-Fire 810	Biolegend	SA011F11; Cat. # 149053
Anti-Mouse CXCR5 BV650	Biolegend	L138D7; Cat. # 145517
Anti-Mouse CXCR6 BV711	Biolegend	SA051D1; Cat. # 151111
Anti-Mouse F4/80 BV480	BD Biosciences	T45-2342; Cat. # 565635
Anti-Mouse FoxP3 Pacific Blue	Biolegend	MF-14; Cat. # 126410
Anti-Mouse FR4 BUV563	BD Biosciences	12A5; Cat. # 748996
Anti-Mouse IA/IE (MHCII) BV480	BD Biosciences	M5/114.15.2; Cat. # 566088
Anti-Mouse Ki67 PE-Cy7	Biolegend	16A8; Cat. # 652426
Anti-Mouse KLRG1 PE-Fire 810	Biolegend	2F1/KLRG1; Cat. # 138437
Anti-Mouse PD-1 PE/Dazzle 594	Biolegend	RMP1-30; Cat. # 109115
Anti-Mouse Thy1.2 BV421	Biolegend	30-H12; Cat. # 105341
**Chemicals and Reagents**		
ACK Lysing buffer	Gibco	Ref. # A10492-01
Collagenase Millipore	Sigma	Cat. # 11088882001
DNAse Millipore	Sigma	Cat. # DN25-5G
DPBS	Corning	Cat. # 20-030-CV
EDTA	Corning	Cat. # 46-034-CI
Fetal Bovine Serum	GeminiBio	Cat. # 100-106
Fixable Viability Dye Zombie NIR	Biolegend	Cat. # 423106
FoxP3 Fix/Perm Concentrate	Cytek	Cat. # TNB-1020-L050
FoxP3 Fix/Perm Diluent	Cytek	Cat. # TNB-1022-L160
Flow Cytometry Perm Buffer	Cytek	Cat. # TNB-1213-L150
GolgiPlug	BD Biosciences	Cat # 555029
GolgiStop	BD Biosciences	Cat # 554724
IMDM	Gibco	Ref # 122440-053
Penicillin/Streptomycin/L-glutamine	GeminiBio	Cat# c400-110
Permeablization Buffer	Invitrogen	Cat # 00-8333-56
RPMI 1640	Corning	Cat #10-040-CM
**Software and Algorithms**		
FlowJo v 10.7 software	Tree Star	`https://www.flowjo.com`
Prism v 9.4.0 software	GraphPad	`www.graphpad.com`
CmdStan v 2.34 software	Stan	`www.mc-stan.org`
Pyro v 1.9.0 software	Pyro	`https://pyro.ai`

Table S2: Reagents and resources

bioRxiv preprint doi: https://doi.org/10.1101/2024.07.08.602409; this version posted February 25, 2025. The copyright holder for this preprint (which was not certified by peer review) is the author/funder, who has granted bioRxiv a license to display the preprint in perpetuity. It is made available under aCC-BY 4.0 International license.

Parameter	Description	Prior	Hyper-parameters
$\lambda_{E,i}$	Initial net loss rate of cluster i	$\lambda_{E,i} \sim \mathcal{N}(\mu_{\lambda_E}, \sigma_{\lambda_E})$	$\mu_{\lambda_E} \sim \mathcal{N}(0,1)$, $\sigma_{\lambda_E} \sim \mathrm{HalfNorm}(1)$
$\lambda_{L,i}$	Long-term net loss rate of cluster i	$\lambda_{L,i} \sim \mathcal{N}(\mu_{\lambda_L}, \sigma_{\lambda_L})$	$\mu_{\lambda_L} \sim \mathcal{N}(0,1)$, $\sigma_{\lambda_L} \sim \mathrm{HalfNorm}(1)$
u	Rate at which net loss rate λ changes from λ_E to λ_L	$u \sim \mathrm{HalfNorm}(1)$	-
Q_{ij}	Rate of differentiation from cluster j to i	$Q_{ij} \sim \mathrm{Exp}(\mu_Q^{-1})$	$\mu_Q \sim \mathrm{Exp}(100)$
$X_{0,i}$	Initial size of cluster i	$X_{0,i} \sim \mathrm{LogNorm}(0, 10)$	-
σ_M	Scale parameter for likelihood of T cell numbers	$\sigma_M \sim \mathrm{Exp}(1)$	-
τ_K	Dispersion parameter for likelihood of cluster size	$\tau_K^{-1} \sim \mathrm{Exp}(10^3)$	-
Specialized priors for the integrated approach			
Q_{ij}	Rate of differentiation from cluster j to i	$Q_{ij} \sim \mathrm{Exp}(\mu_Q^{-1} \exp(\alpha_Q(D_{ij} - \bar{D}))$	$\mu_Q \sim \mathrm{Exp}(100)$, $\alpha_Q \sim \mathrm{HalfNorm}(1)$

Table S3: Prior distributions for the Bayesian model. Normal distributions are parameterized with scale parameters instead of variance. The half-normal distribution is denoted $\mathrm{HalfNorm}(\sigma)$, and the log-normal distribution $\mathrm{LogNorm}(\mu, \sigma)$. The prior for Q_{ij} in the integrated approach is informed by the distance $D_{ij} = \|\mu_i - \mu_j\|_2$ between GMM cmponent i and j in the latent space.
